# Population genetics of group B *Streptococcus* from maternal carriage in an ethnically diverse community in London

**DOI:** 10.3389/fmicb.2023.1185753

**Published:** 2023-05-18

**Authors:** Dorota Jamrozy, Guduru Gopal Rao, Theresa Feltwell, Theresa Lamagni, Priya Khanna, Androulla Efstratiou, Julian Parkhill, Stephen D. Bentley

**Affiliations:** ^1^Parasites and Microbes Programme, Wellcome Sanger Institute, Hinxton, United Kingdom; ^2^Department of Microbiology, Northwick Park Hospital, London North West University Healthcare NHS Trust, London, United Kingdom; ^3^Faculty of Medicine, Imperial College, London, United Kingdom; ^4^Cambridge Institute for Medical Research, School of Clinical Medicine, University of Cambridge, Cambridge, United Kingdom; ^5^World Health Organization Collaborating Centre for Diphtheria and Streptococcal Infections, UK Health Security Agency, London, United Kingdom; ^6^Department of Veterinary Medicine, University of Cambridge, Cambridge, United Kingdom

**Keywords:** group B *Streptococcus*, *Streptococcus agalactiae*, maternal carriage, maternal immunization, serotypes, clonal complexes, antimicrobial resistance, vaccine

## Abstract

**Introduction:**

Maternal immunization against Group B *Streptococcus* (GBS) has the potential to significantly reduce the burden of neonatal GBS infections. Population genetics of GBS from maternal carriage can offer key insights into vaccine target distribution.

**Methods:**

In this study we characterized the population structure of GBS isolates from maternal carriage (*n* = 535) in an ethnically diverse community in London, using whole genome sequencing.

**Results:**

The isolates clustered into nine clonal complexes (CCs) but the majority (95%) belonged to five lineages: CC1 (26%), CC19 (26%), CC23 (20%), CC17 (13%) and CC8/10 (10%). Nine serotypes were identified, the most common were serotypes III (26%), V (21%), II (19%) and Ia (19%). Other serotypes (Ib, IV, VI, VII, IX) represented less than 10% of all isolates each. Intra-lineage serotype diversity was observed in all major CCs but was highest in CC1, which revealed nine serotypes. Nearly all isolates (99%) carried at least one of the four alpha family protein genes (*alpha*, *alp1*, *alp23*, and *rib*). All isolates were susceptible to penicillin. We found 21% and 13% of isolates to be resistant to clarithromycin and clindamycin, respectively. Prevalence of macrolide-lincosamide-streptogramin B (MLS_B_) resistance genes was 22% and they were most common in CC19 (37%) and CC1 (28%), and isolates with serotypes V (38%) and IV (32%). We identified some associations between maternal ethnicity and GBS population structure. Serotype Ib was significantly less common among the South Asian compared to Black women (S. Asian: 3/142, Black: 15/135, *p* = 0.03). There was also a significantly lower proportion of CC1 isolates among the White other (24/142) in comparison to Black (43/135) and S. Asian (44/142) women (*p* = 0.04). We found a significantly higher proportion of CC17 isolates among the White other compared to S. Asian women (White other: 32/142, S. Asian: 10/142, *p* = 0.004).

**Conclusion:**

Our study showed high prevalence of GBS vaccine targets among isolates from pregnant women in London. However, the observed serotype diversity in CC1 and high prevalence of MLS_B_ resistance genes in CC19 demonstrates presence of high risk lineages, which might act as a reservoir of non-vaccine strains and antimicrobial resistance determinants.

## 1. Introduction

Group B *Streptococcus* (GBS) is a commensal bacterium that can colonize human gastrointestinal and genital tracts. Maternal carriage of GBS during pregnancy is a risk factor for development of invasive disease in the newborn. Worldwide GBS maternal colonization prevalence is estimated at 18%, with regional variation ranging from 11% to 35% (Russell et al., 2017). Around 30%–70% of babies born to mothers that carry GBS become colonized themselves, out of which about 1%–3% will develop an invasive disease (Melin, 2011). Depending on the time when the disease occurs during a neonatal period, it will be classified as either early onset (EO, at 0–6 days of life) or late-onset (LO, at 7–89 days of life). EO GBS disease can be prevented through intrapartum antibiotic prophylaxis (IAP), which involves intravenous administration of antibiotics before delivery (Schuchat et al., 1996). This prevention strategy has been implemented in a number of high-income countries, and in many cases, it resulted in a decline of EO disease incidence (Schrag et al., 2000; Lopez Sastre et al., 2005; Darlow et al., 2016). However, there are limitations to IAP as a key strategy for prevention of GBS infant disease. Implementation of IAP policy has been sparse in low- and middle-income countries, where it is less feasible due to limited access to healthcare and high proportion of home births (Le Doare et al., 2017). Furthermore, IAP has no effect on the incidence of LO disease, which has remained stable or increased over time (Schrag et al., 2000; Romain et al., 2017). There is therefore an urgent need to develop alternative strategy for the prevention of neonatal GBS disease and maternal GBS vaccine is the main contender.

Capsular polysaccharide (CPS) is a virulence factor of GBS, involved in the evasion of host’s immune response (Marques et al., 1992). Ten CPS serotypes have been identified in GBS (Ia, Ib, and II-IX) and the most common are Ia, Ib, II, III, and V, estimated to account for 98% of GBS isolates from maternal colonization and 97% of infant GBS disease cases globally (Madrid et al., 2017; Russell et al., 2017). Sufficient levels of maternal anti-CPS antibodies can protect the infant against GBS disease on a serotype-specific basis (Baker and Kasper, 1976) and there is currently a number of multi-valent CPS conjugate vaccines under clinical evaluation (Carreras-Abad et al., 2020). However, implementation of a CPS-based vaccine against carriage of GBS during pregnancy has the potential to exert selective pressure on GBS population, which might lead to serotype switching, serotype replacement and expansion of non-vaccine GBS types (Gladstone et al., 2015). Alternative protein-based vaccine formulations are also under development, which includes a vaccine based on the N-terminal domains of four GBS surface proteins from the alpha protein family (Alpha C, Alp1, Alp2/3, and Rib; Brokaw et al., 2022).

We need to better understand the GBS molecular epidemiology, in particular vaccine target prevalence, to determine vaccine coverage in different countries and regions. In addition, there is also a need to learn more about relationship between GBS serotypes and GBS lineages as well as carriage of antimicrobial resistance (AMR) genes to identify high-risk lineages that may rise in frequency following CPS-specific immunizations. Here, we describe the population genetics of GBS isolates derived from maternal carriage in London, based on whole-genome sequence analysis. We report the population structure, distribution of serotypes, alpha family protein genes and prevalence of AMR determinants. We also describe association between GBS population structure and maternal ethnicity.

## 2. Materials and methods

### 2.1. Bacterial isolates

GBS from maternal carriage was isolated from recto-vaginal swabs from pregnant women at 35–37 weeks of gestation that were collected between March 2014 and December 2015 during a screening program for the prevention of early-onset GBS infection implemented in London North West University Healthcare NHS Trust, United Kingdom, as described previously (Rao et al., 2017). A total of 656 isolates were randomly selected for sequencing. Subsequently, 20 samples were removed from further analysis following initial post-sequencing quality check. After further data analysis and sample metadata review, a further 101 samples were removed due to being identified as potential duplicates. A total of 535 GBS isolates were selected for the final analysis. Antibiotic susceptibility to penicillin, clarithromycin, and clindamycin was determined using the disc diffusion method as recommended by the British Society of Antimicrobial Chemotherapy (V.12 January 2013).

### 2.2. Whole-genome sequencing

Bacterial genomic DNA was extracted using Qiagen’s QIAcube HT system. Library construction was carried out using ‘NEB Ultra II custom kit’ on an Agilent Bravo WS automation system. Whole-genome sequencing was performed on the Illumina HiSeq 2000 platform with 125 bp paired-end reads. Annotated assemblies were produced using a pipeline described previously (Page et al., 2016a). For each sample, sequence reads were used to create multiple assemblies using VelvetOptimiser v2.2.5^1^ and Velvet v1.2 (Zerbino and Birney, 2008). An assembly improvement step was applied to the assembly with the best N50 and contigs were scaffolded using SSPACE (Boetzer et al., 2011), with sequence gaps filled using GapFiller (Boetzer and Pirovano, 2012). Automated annotation was performed using PROKKA v1.11 (Seemann, 2014) and a *Streptococcus*-specific database from RefSeq (Pruitt et al., 2012).

### 2.3. Molecular typing

Multi-locus sequence typing (MLST) was performed using SRST2 (Jones et al., 2003; Inouye et al., 2014). Isolates were assigned to a CC group based on MLST and cluster assignment from fastbaps v1.0.6 (Tonkin-Hill et al., 2019). The fastbaps clustering was performed using the “symmetric” prior and a core gene alignment that was generated with panaroo v1.3.0 (Tonkin-Hill et al., 2020). The majority of isolates were grouped into CC based on clusters from level 1. A heterogenous subset of isolates, which represented distinct lineages was grouped into a single cluster and those isolates were separated further based on clustering using the “optimise symmetric” prior levels 1 and 2. The results of fastbaps clustering are provided in Supplementary Table 1. CPS serotype was determined by *in silico* PCR using previously described primers for the detection of serotypes Ia, Ib, and II-IX (Poyart et al., 2007; Kong et al., 2008). Carriage of AMR genes was checked with SRST2 (Inouye et al., 2014) using the ResFinder database (Zankari et al., 2012), supplemented with sequences of GBS resistance determinants described by Metcalf et al. (2017). PBP2x transpeptidase typing was performed using cd-hit v4.8.1 (Li and Godzik, 2006) and a GBS PBP2x reference database.^2^

### 2.4. Phylogenetic reconstructions

The phylogeny of all isolates was reconstructed based on a core gene alignment that was generated with panaroo v1.3.0 (Tonkin-Hill et al., 2020). Single-nucleotide polymorphisms were identified using SNP-sites (Page et al., 2016b). The maximum likelihood (ML) phylogenetic tree was generated with IQ-TREE (Nguyen et al., 2015) v1.6.12 based on a standard model selection. To reconstruct the phylogeny of each major CC, a whole-genome alignment was generated using a reference-based approach. For this, sequence reads were mapped against CC-specific GBS reference genomes: SS1 (CC1; Flores et al., 2015), Sag37 (CC10), NGBS128 (CC17; Teatero et al., 2016), H002 (CC19; Wang et al., 2015) and NEM316 (CC23; Glaser et al., 2002), using SMALT v0.7.4^3^ Single-nucleotide polymorphisms (SNPs) were called using SAMtools v1.6 (Li et al., 2009) and bcftools v1.6, and identified using SNP-sites (Page et al., 2016b). Alignment positions with >5% uncalled variants were filtered out. Each alignment was screened for variable sites associated with recombination using Gubbins v3.2.1 which were removed (Croucher et al., 2014). ML phylogenetic tree of each CC was generated with IQ-TREE (Nguyen et al., 2015) v1.6.12 based on a standard model selection. The ML phylogenies were partitioned using fastbaps based on a “baps” prior (Tonkin-Hill et al., 2019). Pairwise SNP distances were calculated using the pairwise_difference_count script^4^ on the CC-specific whole-genome alignments.

### 2.5. Statistical analysis

The significance of difference in the frequency of each serotype, CC, alpha family protein genes, and phylogenetic clusters among maternal ethnic groups was analyzed with the Fisher’s exact test. If a statistically significant difference was found, this was followed up with a post-hoc pairwise Fisher’s exact tests with FDR correction. The significance threshold level was set at 0.05. All statistical analyses were performed in RStudio Version 2022.07.2 + 576.

## 3. Results

### 3.1. Overview of GBS population structure, serotypes, alpha family protein genes, and AMR phenotypes and genotypes

MLST revealed 82 distinct STs (Supplementary Table 1). The most common was ST1 (*n* = 85, 16%), followed by ST23 (*n* = 67, 13%), ST17 (*n* = 58, 11%), ST19 (*n* = 57, 11%), and ST28 (*n* = 45, 8%). Of the 82 unique STs, 44 (54%) were represented by a singleton. A total of 506 of the isolates (95%) clustered into five major GBS CCs: CC19 (*n* = 139, 26%), CC1 (*n* = 137, 26%), CC23 (*n* = 109, 20%), CC17 (*n* = 70, 13%), and CC8/10 (*n* = 51, 10%). Less prevalent CCs were also identified (CC22, CC26, CC130, and CC452), while four isolates represented singletons (ST889, ST1438, ST1639, and ST1642). The ST composition of each CC is provided in Supplementary Table 2. The phylogeny of all isolates was reconstructed based on a core gene (genes found in at least 99% of the isolates) alignment, which consisted of 1,631 genes and contained 58,688 SNP sites. The CC assignment was plotted on a phylogeny to confirm concordance between CC grouping and the phylogenetic tree (Supplementary Figure 1).

Analysis of serotype distribution revealed that 95% of isolates (*n* = 509) belonged to one of six predominant serotypes (Ia, Ib, II, III, IV, and V). The most common was serotype III (*n* = 139, 26%), followed by serotypes V (*n* = 112, 21%), II (n = 104, 19%), Ia (*n* = 103, 19%), Ib (*n* = 32, 6%), and IV (*n* = 19, 4%). The remaining isolates belonged to rare serotypes that consisted of serotype VI (*n* = 13, 2%), VII (*n* = 3, 1%), and IX (*n* = 10, 2%). There were no isolates with unknown serotype based on genomic analysis.

The GBS isolates were also screened for the presence of genes encoding the alpha family proteins (Alp1, Alp2/3, Alpha C, and Rib), which are included in the protein vaccine candidate GBS-NN/NN2 (Brokaw et al., 2022). A total of 527 (99%) isolates were positive for an alpha family protein gene, with majority carrying a single alpha gene only (*n* = 522, 98%). The most prevalent was *rib* (*n* = 209, 39%), followed by *alp1* (*n* = 114, 21%), *alpha* (*n* = 106, 20%), and *alp2*/3 (*n* = 103, 19%).

Isolates were analyzed for presence of genetic variants associated with AMR. Of particular concern is emergence of isolates with reduced susceptibility to beta-lactams. All GBS isolates in this collection were sensitive to penicillin based on a disc diffusion method. Nevertheless, we analyzed variation in the PBP2x transpeptidase sequence, which is the most common mechanism associated with reduced beta-lactam susceptibility in GBS, using a previously developed PBP2x type database (Metcalf et al., 2017; Kobayashi et al., 2021). The majority of isolates (*n* = 528, 99%) carried one of the four dominant PBP2x transpeptidase types: PBP2x-1 (*n* = 316, 59%), PBP2x-5 (*n* = 109, 20%), PBP2x-2 (*n* = 70, 13%), and PBP2x-4 (*n* = 33, 6%; Supplementary Table 1), none of which has been associated with reduced susceptibility to beta-lactams. A single isolate carried PBP2x-39, which contains a G398A substitution, that has been observed among isolates with reduced beta-lactam susceptibility (Kobayashi et al., 2021). The beta-lactam phenotype of this isolate was re-tested, but it was found to be penicillin and cefoxitin susceptible (E test, 0.094 and 0.125 mg/L, respectively). Five isolates carried novel PBP2x types (four novel variants detected in total), which have not yet been described in a context of beta-lactam non-susceptibility.

Also of concern is an increase in prevalence of resistance to second-line antimicrobials such as macrolides and lincosamides. All isolates were tested for susceptibility to clarithromycin and clindamycin. A total of 121 isolates (23%) were resistant to at least one of these antibiotics (112 [21%] resistant to clarithromycin and 68 [13%] to clindamycin), with 59 (11%) isolates resistant to both (Supplementary Table 1). A total of 120 (22%) carried at least one macrolide-lincosamide-streptogramin B (MLS_B_) resistance gene (*ermA*, *ermB*, *ermT*, *lnuB, lsaC, lsaE*, *mefA*, *mphC*, *msrA,* or *msrD*; Supplementary Table 1). The most prevalent was *ermB* (*n* = 55, 10%), followed by *ermA* (*n* = 39, 7%) and *mefA* (*n* = 27, 5%), which was invariably co-carried with *msrD* (*n* = 27, 5%). There was some discordance of genotype–phenotype resistance results. Of the 121 isolates resistant to clarithromycin and/or clindamycin, 12 (10%) did not carry a known MLS_B_ resistance determinant. Also, 11 out of the 122 isolates that carried a MLS_B_ resistance gene were found to be clarithromycin and clindamycin susceptible.

Human-associated GBS isolates often show a high prevalence of tetracycline resistance genes due to a clonal expansion of GBS lineages that acquired mobile elements carrying *tet* determinants (Da Cunha et al., 2014). In this study, 459 (86%) isolates carried at least one tetracycline resistance gene. The majority of *tet* positive isolates had *tetM* (*n* = 423, 79%), of which six also carried *tetO*. A smaller subset carried *tetO* only (*n* = 33, 6%), while a single isolate had *tetS* gene (Supplementary Table 1).

### 3.2. Pairwise SNP differences and occurrence of clonally related isolates

Transmission of GBS is usually described in a context of mother to baby transfer and there is limited data on transmission of GBS in other settings. We have therefore measured the genetic relatedness among the maternal carriage isolates within this collection by calculating the pairwise SNP distances between isolates within each major CC.

A recent study describing genomic analysis of invasive GBS cases from infants, determined a cut-off of ≤5 SNP difference for identifying disease clusters and inferring horizontal transmission (Collin et al., 2021). We applied this cut-off to screen our dataset for occurrence of closely related carriage strains. We identified 11 pairs, which included one cluster of three isolates, with a pairwise SNP difference ≤ 5 SNP (3 pairs with 0 SNP difference, 4 pairs with 1 SNP difference, 3 pairs with 2 SNP difference, and 1 pair with 3 SNP difference). The isolates represented CC1, CC8/10, and CC19. In all cases, isolates were collected on different days, on average 134 days apart. In a majority of cases, isolates in a pair were collected from women of different ethnicity (7/11) and women living in the same borough (7/11).

### 3.3. Associations between GBS population structure, genotypes, and maternal epidemiological data

**Figure 1:**
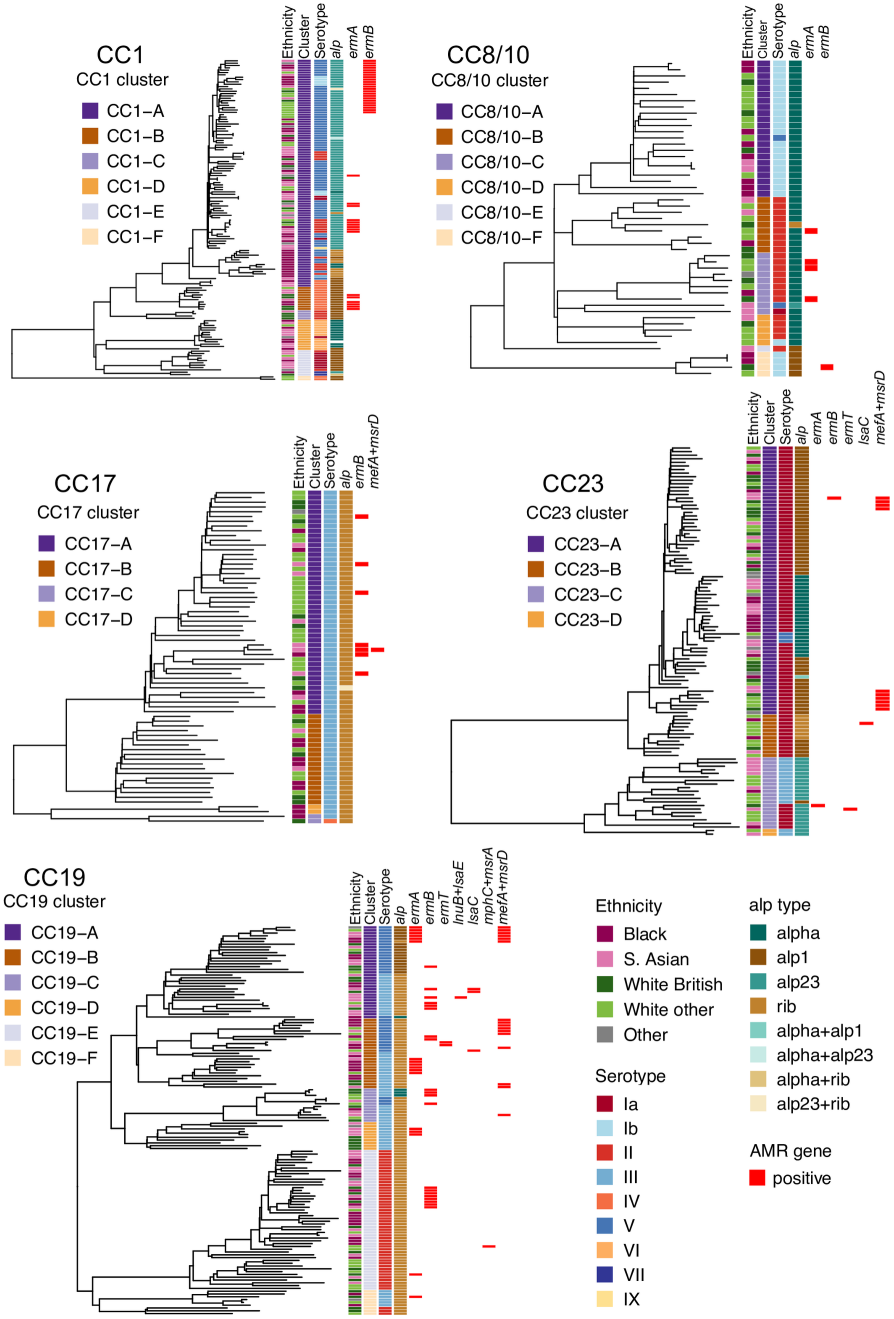
Phylogenetic trees of the five main GBS lineages identified in this study: CC1, CC8/10, CC17, CC19, and CC23. Each tree is annotated with color strips representing (from left): maternal ethnicity, CC-specific cluster ID, serotype, alpha family protein gene, and AMR gene carriage.

To define and analyze the intra-CC population structure, a phylogenetic tree of each major CC was reconstructed and clustered into distinct sub-lineages by partitioning the tree. Based on this, the most heterogeneous lineages were CC1, CC8/10, and CC19, with each clustering into six distinct sub-lineages. The phylogeny of CC17 and CC23 clustered into four sub-lineages. Each tree was annotated with maternal ethnicity, cluster ID, serotype, alpha family proteins, and AMR gene carriage (Figure 1).

#### 3.3.1. GBS population structure and distribution of serotype and alpha family protein genes

**Figure 2:**
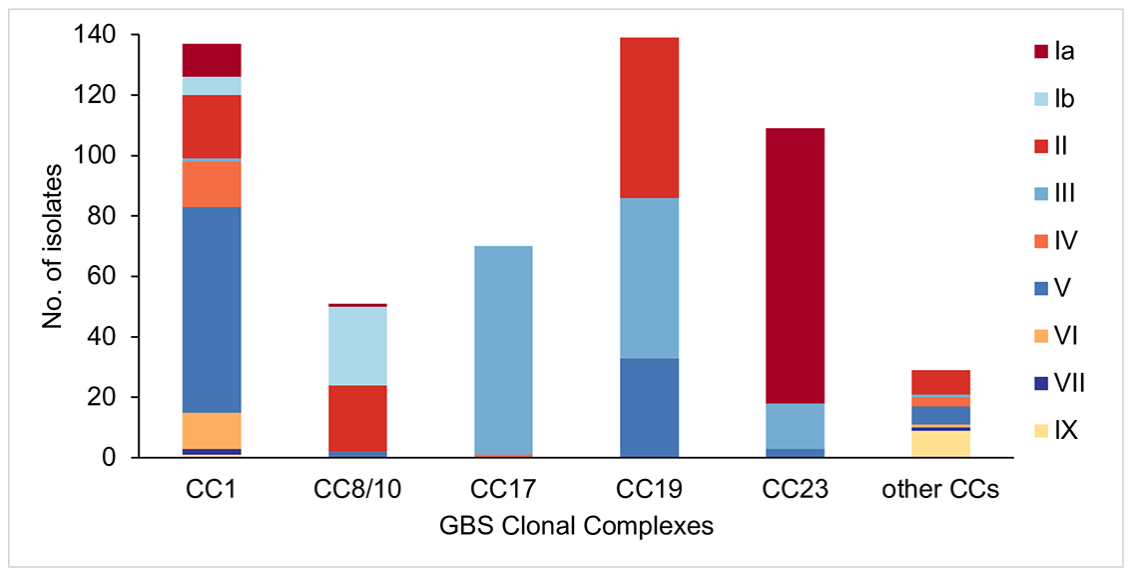
Serotype distribution among the main GBS CCs identified in this study.

The five main GBS lineages showed a varying level of intra-lineage serotype diversity (Figure 2). The lowest serotype heterogeneity was observed in CC17 and CC23. All but one CC17 isolates carried serotype III (*n* = 69, 99%). A single CC17 isolate had serotype IV. The isolate was from ST291 and belonged to a low prevalence cluster CC17-C, which contained only one other isolate that was ST17 and serotype III. The majority of CC23 isolates belonged to serotype Ia (*n* = 91, 83%). The dominant CC23 cluster, CC23-A, had predominantly serotype Ia but also contained three isolates with serotype V (ST24 and ST498). Isolates with the same serotype were clonally related and formed sub-clusters within CC23-A. Serotype heterogeneity was also observed among isolates from a cluster CC23-C, which consisted of sub-clusters of isolates with either serotype III or Ia.

No single serotype was dominant among CC19 isolates that showed three different serotypes: II (*n* = 53, 38%), III (*n* = 53, 38%), and V (*n* = 33, 24%). Serotype heterogeneity occurred in four out of the six CC19 clusters. Clusters CC19-A, CC19-B, and CC19-C consisted of serotype III and V sub-clusters. Clusters CC19-A and CC19-B showed a nearly equal proportion of serotype III and V isolates, while cluster CC19-C mostly consisted of serotype III. The smallest cluster CC19-F consisted of two sub-clusters of isolates with either serotype II or III.

Four serotypes were identified in isolates from CC8/10. The majority of isolates were either serotype: Ib (*n* = 26, 51%) or II (*n* = 22, 43%), with the remining few carrying either serotype Ia (*n* = 1, 2%) or V (*n* = 2, 4%). Three of the five CC8/10 clusters (excluding a singleton with own cluster ID) showed serotype heterogeneity. In each cluster most isolates shared the same serotype, with only a sporadic occurrence of singletons with a different serotype.

Isolates from CC1 were associated with nine distinct serotypes. The most common was serotype V found in half of CC1 isolates (*n* = 68, 50%). Other common serotypes were: II (*n* = 21, 15%), IV (*n* = 15, 11%), VI (*n* = 12, 9%), and Ia (*n* = 11, 8%). The remaining serotypes (Ib, III, VII, and IX) were found in less than 5% of CC1 isolates each. Three out of the six CC1 clusters showed serotype heterogeneity. Each of these clusters had a single dominant serotype and presence of isolates with a different serotype was associated with minor sub-clusters or singletons. The highest serotype diversity was displayed by the dominant cluster CC1-A, which contained seven different serotypes.

Isolates from the other, less prevalent CCs were associated with a single serotype only. All of CC22 isolates were serotype II, CC26—serotype V, CC130—serotype IX, and CC452—serotype IV.

**Figure 3:**
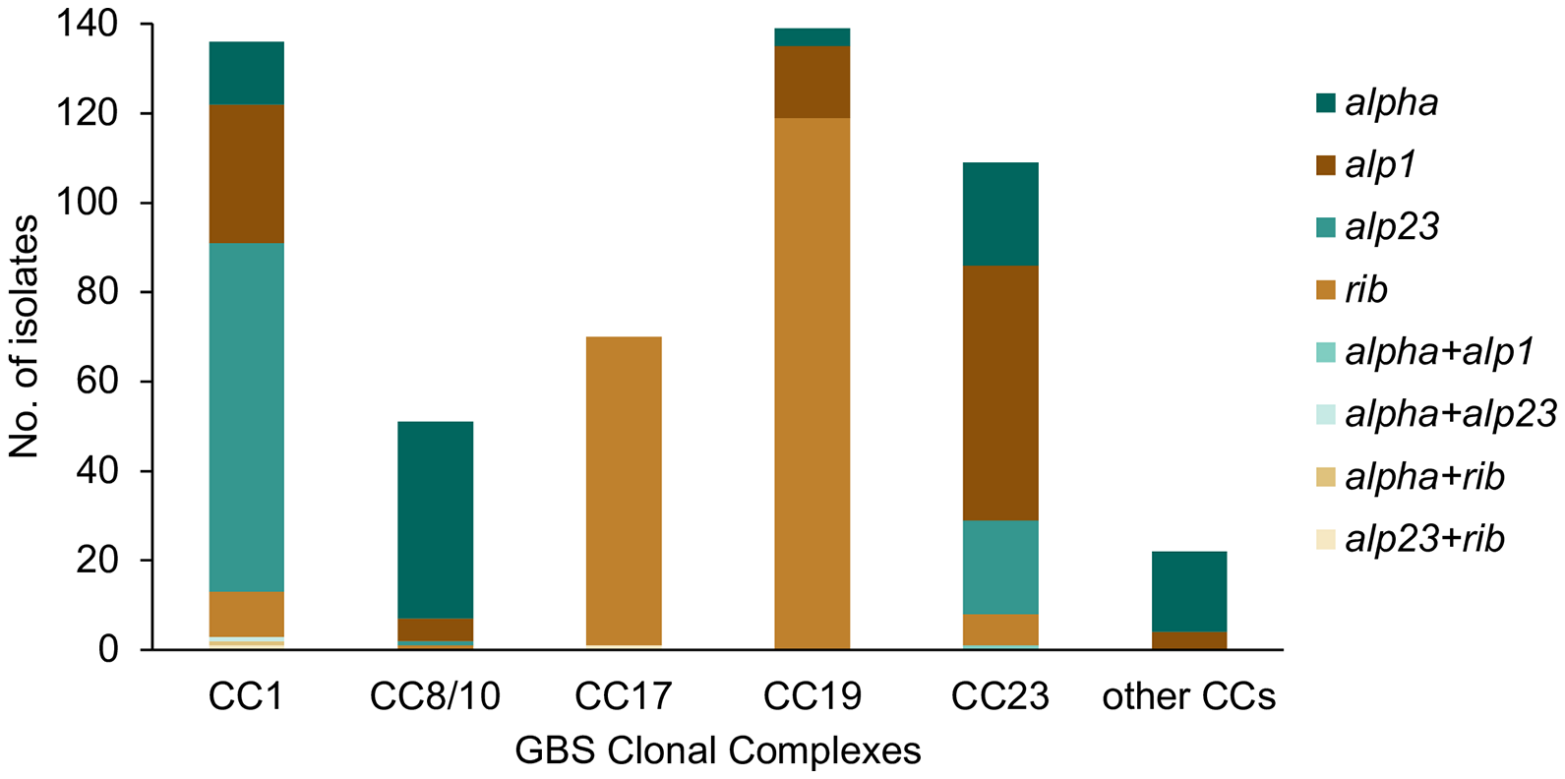
Alpha family protein gene distribution among the main GBS CCs identified in this study. Most isolates carried a single alpha family protein type (*alpha*, *alp1*, *alp23,* or *rib*). For isolates carrying more than one alpha family protein type, the observed co-carriage combinations (*alpha* + *alp1*, *alpha* + *alp23*, *alpha* + *rib*, and *alp23* + *rib*) are represented separately on the graph.

All major CCs showed presence of multiple alpha family protein types (Figure 3). The lowest heterogeneity of the alpha family protein genes was observed in CC17 with all isolates carrying the *rib* gene and a single isolate also positive for *alp23*. The *rib* gene was also dominant in CC19 (n = 119, 86%). In CC1 the most common gene was *alp23* (*n* = 80, 58%), in CC8/10 the majority of isolates carried *alpha* (*n* = 44, 86%), while in CC23, the most prevalent was *alp1* (*n* = 58, 53%). Across CC phylogenies, carriage of diverse alpha family protein genes was associated with both clonal distribution as well as sporadic occurrence (Figure 1). Isolates that lacked any of the four alpha family protein types belonged to CC1 (*n* = 1), CC26 (*n* = 6), and ST1438 (*n* = 1).

#### 3.3.2. GBS population structure and distribution of AMR genes

The distribution of PBP2x transpeptidase types was CC-specific. The PBP2x-1 was predominant among CC1 (*n* = 128, 93%), CC8/10 (*n* = 51, 100%), and CC19 (*n* = 136, 98%) isolates. The PBP2x-2 was found in the majority of CC17 isolates (*n* = 69, 99%). The remaining CC17 isolate carried PBP2x-39. Most of CC23 isolates carried PBP2x-5 (*n* = 106, 97%). The five isolates with novel PBP2x transpeptidase types represented various CCs.

The highest prevalence of MLS_B_ resistance genes was found in isolates from CC19 (*n* = 52, 37%) and CC1 (*n* = 38, 28%), and was lower among isolates from CC17 (*n* = 7, 10%), CC23 (*n* = 13, 12%), and CC8/10 (*n* = 5, 10%). Among rare CCs, MLS_B_ resistance genes were observed in CC22 (*n* = 4) and CC26 (*n* = 1). The MLS_B_ resistance genes showed inter-lineage distribution with no gene associated with a single CC.

The distribution of MLS_B_ resistance genes was analyzed further in a context of CC-specific population structure and phylogenetic clustering. CC1 isolates carried *ermA* and *ermB* genes. All CC1 *ermB*-positive isolates formed a single monophyletic sub-cluster within cluster CC1-A. The *ermA* gene was found among multiple minor sub-clusters representing the CC1-A and CC1-B phylogenetic groups. In CC19, *ermA* and *ermB* genes were distributed across the phylogeny and each was associated with various phylogenetic clusters, while the less prevalent genes (*ermT*, *lnuB*, *lsaC, lsaE, mefA,* and *msrD*) were found predominantly in CC19-A and CC19-B groups. In CC8/10, CC17, and CC23, the occurrence of MLS_B_ resistance genes was sporadic with limited or no clonal spread.

We also analyzed association between carriage of the MLS_B_ resistance genes and different serotypes. Carriage of MLS_B_ resistance genes was most prevalent among isolates with serotypes V (*n* = 43, 38%) and IV (*n* = 6, 32%). For serotype V, this was associated predominantly with isolates from CC1 and CC19, whereas for serotype IV, all isolates were from CC1. MLS_B_ resistance genes were less common among isolates with serotype Ia (*n* = 13, 13%), Ib (*n* = 5, 16%), II (*n* = 24, 23%), and III (*n* = 29, 21%), and absent in isolates with serotypes VI, VII, and IX.

Prevalence of *tet* genes varied between CCs and was highest in CC17 (*n* = 68, 97%), followed by CC19 (*n* = 127, 91%), CC8/10 (*n* = 45, 88%), CC23 (n = 96, 88%), and CC1 (*n* = 110, 80%). No *tet* genes were identified in isolates from CC130, CC452, ST1438, and ST1642. For all lineages with a *tet* determinant, the dominant tetracycline resistance gene was *tetM.* A small subset of isolates carried *tetO,* which was most common in CC8/10 (*n* = 9, 18%) and CC19 (*n* = 25, 18%), followed by CC17 (*n* = 5, 7%) and CC23 (*n* = 2, 2%). It was not detected in CC1 isolates. Most isolates with *tetO* lacked *tetM*.

#### 3.3.3. Associations between GBS genotypes and maternal ethnicity

We investigated if there were any associations between the maternal ethnicity and GBS genotypes (serotype, alpha family protein gene carriage, CC, and phylogenetic clusters). There were four main ethnic groups among the women that participated in the GBS screening program: Black (*n* = 135, 25%), South Asian (S. Asian; *n* = 142, 27%), White British (*n* = 93, 17%), and White other (*n* = 142, 27%). The remaining women (*n* = 23, 4%) represented minority groups composed of Mixed, Oriental, and other ethnicities, which were grouped here as Other.

**Figure 4:**
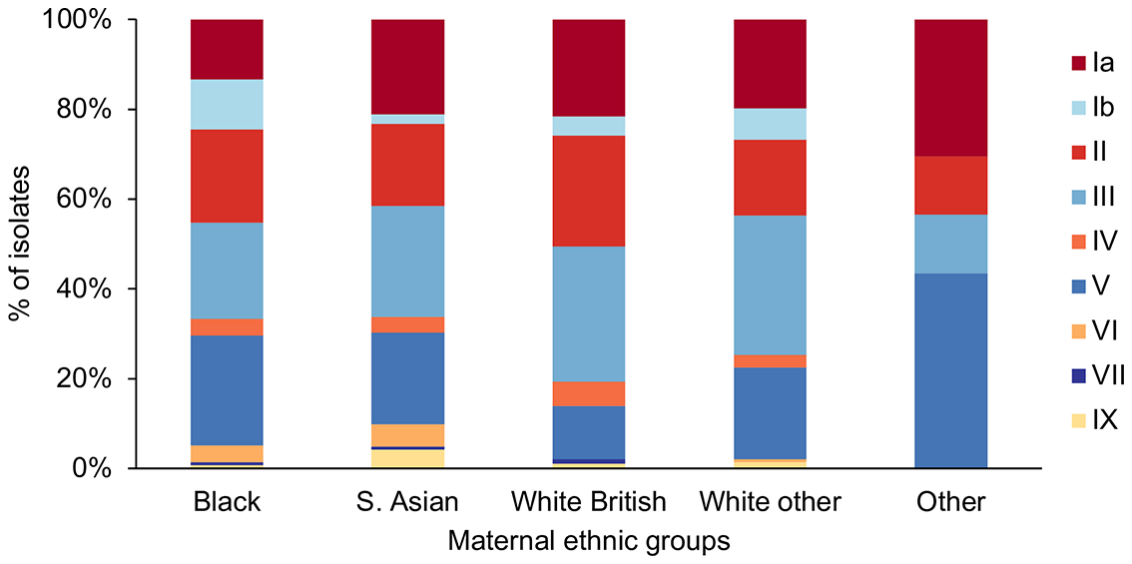
GBS serotype distribution among the different maternal ethnic groups.

We found a significant difference in the frequency of serotype Ib (*p* = 0.019) among the different ethnic groups (Figure 4), as it was significantly less prevalent among the S. Asian women in comparison to Black women (S. Asian: 3/142, Black: 15/135, *p* = 0.03). The White British women showed the lowest prevalence of GBS with serotype V, but it was significant only in comparison to the Other ethnic group (White British: 11/93, Other: 10/23, *p* = 0.01).

**Figure 5:**
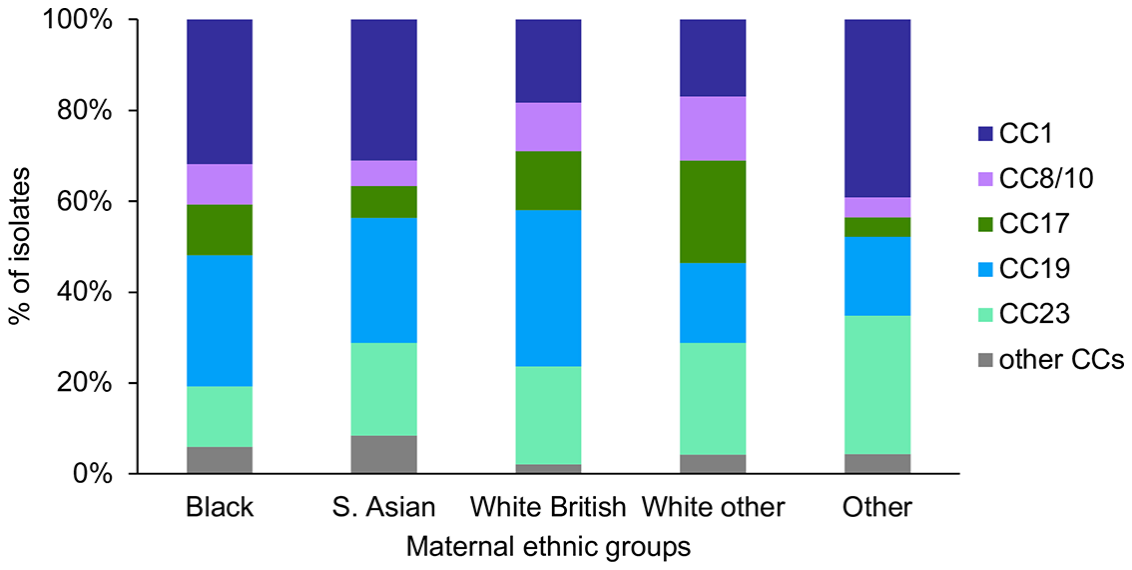
GBS CC distribution among the different maternal ethnic groups.

There was a significant difference in the prevalence of CC1 (*p* = 0.003) and CC17 (*p* = 0.002) among the ethnic groups (Figure 5). Pairwise tests for CC1 distribution showed that there was a significantly higher prevalence of CC1 isolates among the Black (43/135) and S. Asian (44/142) women, in comparison to the White other (24/142) women (*p* = 0.04). There was also a significantly higher proportion of CC17 isolates among the White other in comparison to the S. Asian women (White other: 32/142, S. Asian: 10/142, *p* = 0.004).

**Figure 6:**
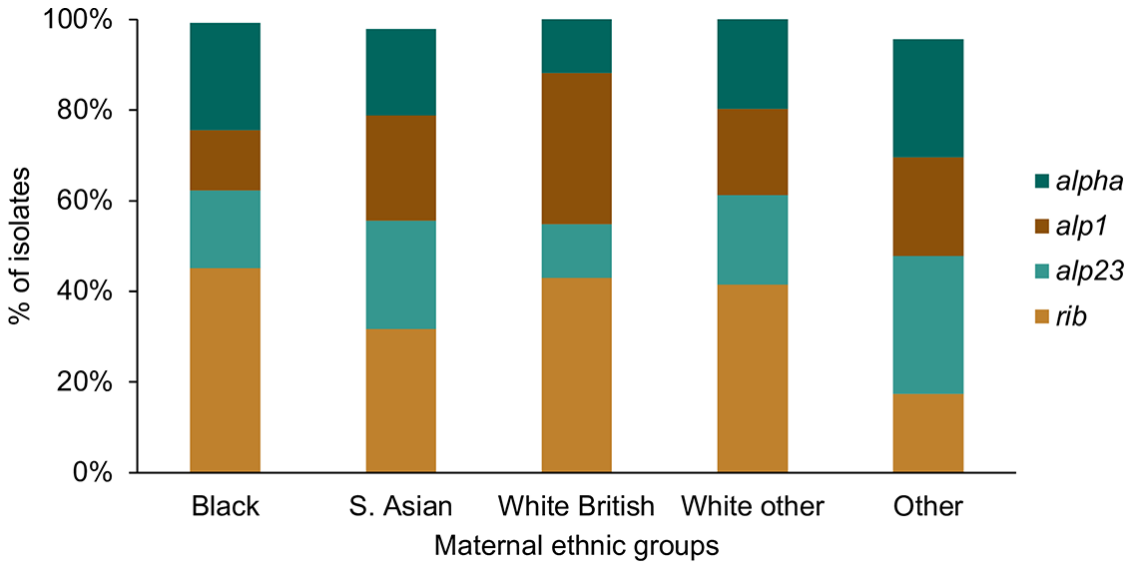
GBS alpha family protein gene distribution among the different maternal ethnic groups.

For the alpha family protein gene carriage, we found a significantly higher prevalence of *alp1* positive GBS among the White British in comparison to the Black women (White British: 31/93, Black: 18/135, *p* = 0.005; Figure 6). The prevalence of GBS without any of the four alpha family protein genes was highest among women from the Other ethnic groups, although this was not statistically significant.

For each of the five major CCs, we also investigated variation in the distribution of isolates from the different phylogenetic clusters between the different maternal ethnic groups. This showed no associations except for a significantly higher prevalence of the CC23-A cluster isolates among CC23 in the White British in comparison to the White other women (White British: 18/20, White other women: 17/35, *p* = 0.03). We also observed that the high frequency of the CC17 isolates among the White other women was largely due to a higher number of isolates from cluster CC17-A in comparison to all other ethnic groups, although it was not statistically significant.

## 4. Discussion

There is an urgent need for an effective maternal GBS vaccine as an alternative to current methods for the prevention of invasive GBS infections in newborn babies. Among key candidates are multivalent CPS conjugate vaccine formulations that would offer protection on a serotype-specific basis. One such vaccine, GBS6, which includes six dominant GBS serotypes (Buurman et al., 2019), would offer a 95% coverage in this maternal population. While maternal GBS immunization has the potential to significantly reduce the global burden of neonatal invasive GBS disease, vaccination against commensal bacteria such as GBS poses certain risks. In most human hosts, GBS is associated with asymptomatic carriage, and its eradication might disrupt commensal microbiota balance, which, in turn, can lead to introduction or expansion of a more pathogenic organism or impact the development of the immune system (McDaniel and Swiatlo, 2016). Furthermore, a selective pressure of a vaccine targeting only selected GBS CPS serotypes might result in expansion of non-vaccine GBS lineages (Gladstone et al., 2015). To better understand and evaluate the latter risk, we need more data on GBS population structure, in particular for isolates from maternal colonization.

The population of GBS from maternal carriage in London correlates with previous studies reporting CCs found among GBS from maternal colonization (Seale et al., 2016; Teatero et al., 2017). The two most common lineages in this collection were CC1 and CC19. Isolates from CC1 demonstrated the highest number of unique STs and the highest serotype heterogeneity. The majority of CC1 isolates were ST1, most of which were serotype V. This GBS genotype has been previously associated with disease in non-pregnant adults (Flores et al., 2015; Lopes et al., 2018). Six other serotypes were also observed among ST1 isolates, which contributed to the observed serotype diversity within CC1. Isolates from CC19 were less heterogeneous than CC1 based on serotype but showed the highest prevalence of MLS_B_ resistance genes. Altogether, the two lineages, CC1 and CC19, require further attention in the context of GBS genomic surveillance as each represents a potential reservoir of high-risk lineages—associated either with non-vaccine serotypes or with high prevalence of AMR genes. Also, an overall widespread distribution of the MLS_B_ resistance genes among the analyzed isolates, suggests a high capacity for dissemination and transfer among various GBS lineages. As such, a further investigation of the mobile genetic elements carrying these resistance genes is warranted.

We found that a protein-based vaccine targeting the alpha family proteins would offer a high coverage within the analyzed maternal population. However, the relatively high intra-linage diversity of the alpha family protein genes observed here, suggests that these genes can undergo recombination, which might impact on their prevalence following a selective pressure of vaccination. We also found a low frequency of isolates that lacked any of the four alpha family protein genes, indicating that non-vaccine types circulate within GBS population and can increase in prevalence following vaccination.

The identification of 11 pairs of closely related isolates (≤5 SNP distance) was surprising. This is indicative of a recent transmission event although the possible route of transmission within this patient cohort is unknown. There are currently limited data on the prevalence of horizontal transmission of GBS, with focus primarily on its role in the acquisition of GBS LO disease among neonates (Jauneikaite et al., 2018). Possible routes of GBS transmission among non-hospitalized adults include sexual and casual contact, although the latter is considered a low risk for GBS transmission (Yamamoto et al., 1999; Manning et al., 2004; Foxman et al., 2006). However, it has also been suggested that oropharyngeal GBS colonization might play a role in GBS transmission, particularly among neonates with GBS LO disease (Roloff et al., 2018). While it is still unclear whether a link between oropharyngeal and rectovaginal colonization exists, oropharyngeal GBS colonization might contribute to horizontal transmission of GBS. Furthermore, a probable horizontal transmission of GBS between pregnant women that was identified in this study aligns with previous reports describing instances of new GBS acquisition during pregnancy, which has implications for the predictive value of antenatal GBS screening (Valkenburg-Van Den Berg et al., 2006; Kwatra et al., 2014).

Serotype diversity within a CC, such as presence of 3 or more different serotypes, was mostly associated with occurrence of distinct serotype sub-populations within phylogenetic clusters. However, single strains with a serotype different to the background population were also observed, in particular in CC1. This was particularly prominent in cluster CC1-A, which was predominantly serotype V but also contained eight other serotypes that were interspersed among serotype V isolates. Some of these may represent recent serotype switching events. However, we also found that CC1 shows an overall high level of genetic diversity with a high number of unique ST. The serotype diversity might further reflect a highly heterogenous population that constitutes CC1, which, in turn, may be associated with historical rather than recent recombination events.

The analysis of associations between maternal ethnicity and GBS population structure showed significant variation in the prevalence of certain serotypes and CCs between some ethnic groups. In particular, isolates from CC1 were more common among Black and S. Asian in comparison to White other women, while CC17 was more prevalent in the White other in comparison to the S. Asian group. The mechanisms of this association are unknown. It is unlikely to be associated with a recent clonal transmission of these lineages among women of the same ethnic background. Although we identified some instances of pairs of clonal isolates within this collection, in majority of cases, the isolates were from women of different ethnic groups. The CC-ethnicity associations may reflect correlations between particular CC and maternal country of birth. For instance, most of the White other women that participated in this study would have an Eastern European background, with majority being either Polish or Romanian (Rao et al., 2017). As such, the observed higher prevalence of CC17 isolates among these women may indicate that maternal colonization with CC17 is more prevalent in these countries. However, a country-specific data on molecular epidemiology of GBS would be required to further verify this.

It is also unclear whether the observed differences in the GBS CC and serotype distribution between the maternal ethnic groups can be correlated with maternal GBS colonization rates and disease incidence. Previous study describing the same patient cohort reported that the GBS colonization was strongly associated with ethnicity with the highest colonization rates observed among women of Black African origin and the lowest in the S. Asian women (Gopal Rao et al., 2019). In this study, we found that GBS serotype Ib was significantly more common among Black in comparison to the S. Asian women, with S. Asian women showing the lowest prevalence of serotype Ib in comparison to all other ethnic groups. Serotype Ib was associated in this collection with CC1 and CC8/10 isolates, and both CC1-Ib and CC8/10-Ib genotypes were observed among the Black ethnic group, indicating that increased prevalence of serotype Ib among Black women is mediated by multiple GBS lineages. However, the significance of this remains unclear. Although it was found most prevalent among Black women, serotype Ib was not a dominant serotype within this ethnic group. Furthermore, a recent systematic review of global molecular epidemiology of GBS found a relatively low prevalence of serotype Ib among maternal GBS carriage or disease isolates (Bianchi-Jassir et al., 2020). Therefore, the potential association between serotype Ib and maternal GBS carriage within certain ethnic groups requires further scrutiny.

Our study was limited by a relatively small sample size, in particular with relation to ascertaining the associations between certain GBS genotypes and maternal ethnicity. Also, due to lack of more detailed demographic data such as maternal country of birth, we could not investigate further the mechanisms behind these associations. Furthermore, the GBS population in this host group might have changed since the time of sample collection, and the presented here distribution of GBS lineages and serotypes might no longer fully represent the circulating GBS clones.

Further genomic surveillance of GBS from maternal carriage and neonatal disease is required to characterize the genetic diversity of GBS population in different countries and regions. Analysis of GBS isolates from pregnant women in North-West London showed that while the majority carried serotypes that are included in the GBS6 vaccine, some lineages such as CC1 show high serotype diversity and might act as a reservoir of non-vaccine strains. Better understanding of GBS population diversity prior to vaccine implementation will help us to monitor any changes that may subsequently arise and identify in advance high-risk lineages.

## Data availability statement

The datasets presented in this study can be found in online repositories. The names of the repository/repositories and accession number(s) can be found at: https://www.ebi.ac.uk/ena, PRJEB20117.

## Ethics statement

Ethics approval and written informed consent for participation was not required for the sample collection study (Rao et al., 2017) and the subsequent sequencing and analysis of the isolates because the samples were collected during routine care and anonymized. Maternal demographics were routinely collected as part of maternity care and the data presented in this paper do not identify individual women (Gopal Rao et al., 2019).

## Author contributions

GG, TL, AE, and JP conceived the study. TF and PK performed the laboratory work. DJ analyzed the data with guidance from SDB. DJ drafted the manuscript. All authors contributed to the article and approved the submitted version.

## Funding

This work was supported by the London North West Healthcare Charity, and was funded in part by the Wellcome Trust [Grant number 206194]. For the purpose of Open Access, the author has applied a CC BY public copyright licence to any Author Accepted Manuscript version arising from this submission.

## Conflict of interest

The authors declare that the research was conducted in the absence of any commercial or financial relationships that could be construed as a potential conflict of interest.

## Publisher’s note

All claims expressed in this article are solely those of the authors and do not necessarily represent those of their affiliated organizations, or those of the publisher, the editors and the reviewers. Any product that may be evaluated in this article, or claim that may be made by its manufacturer, is not guaranteed or endorsed by the publisher.

## Footnotes

^1^ https://github.com/tseemann/VelvetOptimiser

^2^ https://github.com/BenJamesMetcalf/GBS_Scripts_Reference

^3^ https://www.sanger.ac.uk/science/tools/smalt-0

^4^ https://github.com/sanger-pathogens/bact-gen-scripts/blob/master/pairwise_difference_count.py
