# Supplementary material for: Population genetics of group B Streptococcus from maternal carriage in an ethnically diverse community in London
Source: Front Microbiol. 2023 May 18;14:1185753. doi: 10.3389/fmicb.2023.1185753 (PMC10233156; doi:10.3389/fmicb.2023.1185753)
Supplement: Supplementary file 2 [file Table_2.pdf]

Supplementary Table 2. Sequence type (ST) composition of GBS clonal complexes (CC) identified in this study.

CC	ST composition (number of isolates)
**CC1**	**ST1 (85)**, ST2 (6), ST3 (4), ST4 (5), ST5 (3), ST14 (1), ST136 (4), ST139 (2), ST167 (1), ST196 (6), ST297 (2), ST459 (2), ST796 (2), ST817 (1), ST1435 (1), ST1439 (1), ST1440 (1), ST1624 (1), ST1626 (1), ST1627 (1), ST1630 (1), ST1632 (2), ST1641 (1), ST1643 (1), ST1644 (1), MLST non-typeable (1)
**CC8/10**	ST7 (2), **ST8 (19)**, ST10 (8), ST12 (12), ST255 (4), ST569 (4), ST1441 (1), ST1645 (1)
**CC17**	**ST17 (58)**, ST31 (1), ST95 (1), ST109 (1), ST147 (1), ST291 (2), ST484 (1), ST515 (1), ST1354 (1), ST1625 (1), ST1636 (1), ST1638 (1)
**CC19**	**ST19 (57)**, ST27 (4), ST28 (45), ST110 (7), ST182 (2), ST327 (1), ST335 (6), ST347 (3), ST520 (1), ST529 (8), ST1628 (1), ST1629 (1), ST1631 (1), ST1637 (1), ST1646 (1)
**CC22**	**ST22 (6)**, ST1436 (1)
**CC23**	**ST23 (67)**, ST24 (18), ST88 (4), ST144 (7), ST464 (1), ST477 (1), ST498 (4), ST933 (1), ST978 (2), ST1437 (1), ST1633 (1), ST1634 (1), ST1635 (1)
**CC26**	ST26 (6)
**CC130**	ST130 (9)
**CC452**	ST452 (3)
