# Supplementary material for: Population genetics of group B Streptococcus from maternal carriage in an ethnically diverse community in London
Source: Front Microbiol. 2023 May 18;14:1185753. doi: 10.3389/fmicb.2023.1185753 (PMC10233156; doi:10.3389/fmicb.2023.1185753)

Supplementary Figure 1. Phylogenetic tree of 535 GBS isolates analysed in this study annotated with clonal complex (CC) assignment.

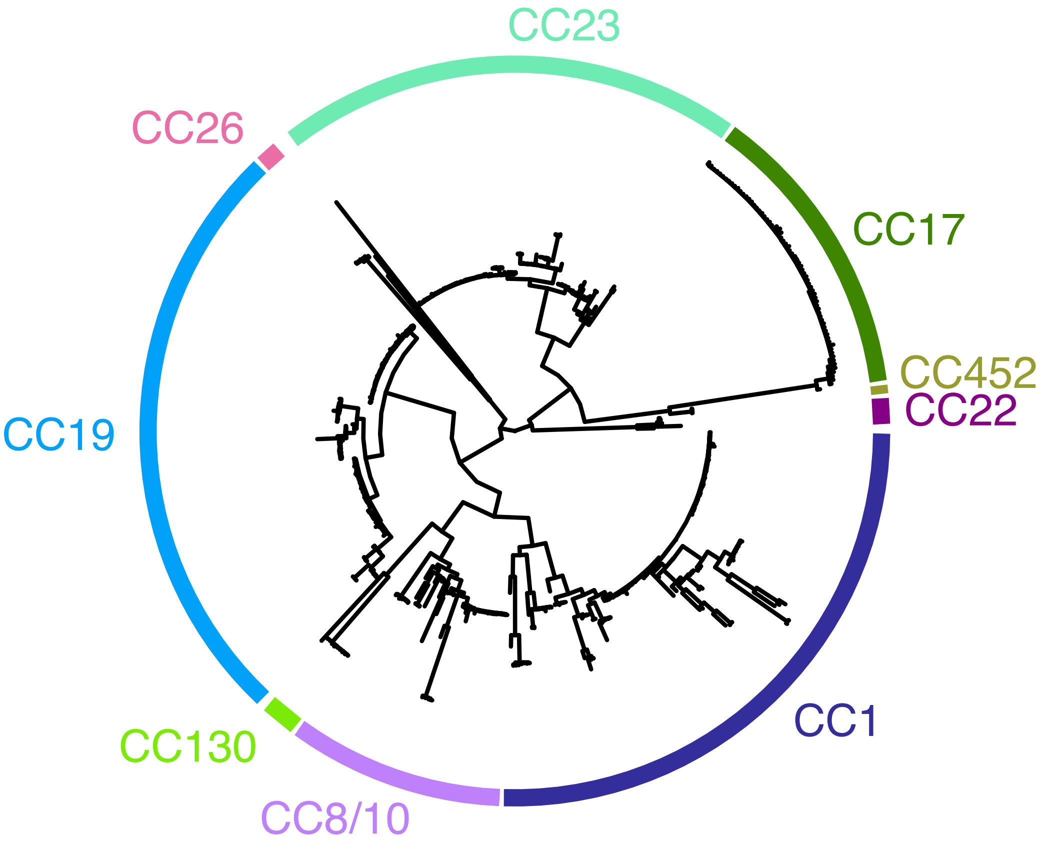

Supplement: Supplementary file 3 [file Image_1.pdf]
